# Active versus resting neuro‐navigated robotic transcranial magnetic stimulation motor mapping

**DOI:** 10.14814/phy2.15346

**Published:** 2022-06-24

**Authors:** Cynthia K. Kahl, Adrianna Giuffre, James G. Wrightson, Adam Kirton, Elizabeth G. Condliffe, Frank P. MacMaster, Ephrem Zewdie

**Affiliations:** ^1^ Department of Psychiatry, Cumming School of Medicine University of Calgary Calgary Alberta Canada; ^2^ Department of Pediatrics, Cumming School of Medicine University of Calgary Calgary Alberta Canada; ^3^ Department of Clinical Neurosciences, Cumming School of Medicine University of Calgary Calgary Alberta Canada; ^4^ Strategic Clinical Network for Neuroscience, Vision, and Rehabilitation Calgary Alberta Canada; ^5^ Strategic Clinical Network for Addictions and Mental Health Calgary Alberta Canada

**Keywords:** motor cortex, motor mapping, non‐invasive brain stimulation, robotic procedures, transcranial magnetic stimulation

## Abstract

Transcranial magnetic stimulation (TMS) motor mapping is a safe, non‐invasive method that can be used to study corticomotor organization. Motor maps are typically acquired at rest, and comparisons to maps obtained during muscle activation have been both limited and contradictory. Understanding the relationship between functional activation of the corticomotor system as recorded by motor mapping is crucial for their use clinically and in research. The present study utilized robotic TMS paired with personalized neuro‐navigation to examine the relationship between resting and active motor map measures and their relationship with motor performance. Twenty healthy right‐handed participants underwent resting and active robotic TMS motor mapping of the first dorsal interosseous to 10% maximum voluntary contraction. Motor map parameters including map area, volume, and measures of map centrality were compared between techniques using paired sample tests of difference and Bland–Altman plots and analysis. Map area, volume, and hotspot magnitude were larger in the active motor maps, while map center of gravity and hotspot locations remained consistent between both maps. No associations were observed between motor maps and motor performance as measured by the Purdue Pegboard Test. Our findings support previous suggestions that maps scale with muscle contraction. Differences in mapping outcomes suggest rest and active motor maps may reflect functionally different corticomotor representations. Advanced analysis methods may better characterize the underlying neurophysiology of both types of motor mapping.

## Graphical abstract

Understanding the relationship between functional activation of the corticomotor system as recorded by transcranial magnetic stimulation (TMS) motor mapping is crucial for their use clinically and in research. The present study utilized robotic TMS paired with personalized neuro‐navigation to examine the relationship between resting and active motor map measures and their relationship with motor performance. Map area, volume, and hotspot magnitude were larger in the active motor maps, while map centre of gravity and hotspot locations remained consistent between both maps, suggesting that they may reflect functionally different corticomotor representations.

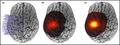

## 1 INTRODUCTION

Transcranial magnetic stimulation (TMS) can be used to safely examine human neurophysiology in vivo and is often used to investigate corticospinal excitability in healthy and clinical populations (Anand & Hotson, 2002; Rossi et al., 2009). TMS can produce high‐resolution cortical maps that are thought to reflect functional corticomotor representations. These maps can be used to examine mechanisms underlying interventional plasticity (Friel et al., 2016), cortical reorganization following trauma or insult (e.g., post‐amputation) (Gagné et al., 2011; Lefaucheur & Picht, 2016), and probe behavior‐induced cortical changes (e.g., motor learning) (Sale et al., 2017). There is also increasing evidence that preoperative TMS motor mapping can improve outcomes from surgery (Krieg et al., 2014; Lefaucheur & Picht, 2016). TMS maps are comparable to those obtained using direct current stimulation, the current gold standard, and possibly more representative of functional motor areas than fMRI (Jeltema et al., 2020). Motor maps have been collected with both the target muscle at rest (Krause et al., 2006; Weiss et al., 2013) and during active contraction (Ngomo et al., 2015). However, the similarities and differences between these techniques are not fully understood, nor is their relationship to motor function. Furthermore, comparison and examination of these techniques are crucial for the continued use of motor mapping clinically and in research.

Motor maps recorded during muscle activation likely differ from those collected at rest. TMS preferentially activates cortical interneurons relaying excitatory inputs to pyramidal neurons (Klomjai et al., 2015). Voluntary muscle contraction increases cortical and spinal excitability, leading to decreased stimulation thresholds (Quartarone et al., 2006; Wassermann, 2002) and increased motor evoked potential (MEP) amplitudes (Buccolieri et al., 2004; Christova et al., 2006; Darling et al., 2006; Di Lazzaro et al., 1998). The change in corticomotor excitability during contraction might result from the effect of muscle afferent activity on corticomotor output (Wilson et al., 1995). It is thought that the increased excitability of the motor pathways due to muscle activation may ‘prime’ either the relevant cortical regions being stimulated, or the relevant motoneurons by decreasing their firing threshold so that they fire earlier in the sequence of descending volleys (Thompson et al., 1991). An alternative interpretation is that increased map size represents the activation of previously silent corticospinal neurons that are responsible for muscle activity (Classen et al., 1998; van de Ruit & Grey, 2016). Motor maps obtained during muscle activation (‘active maps’) may thus differ from those collected while the muscle is at rest (‘resting maps’).

To date, few studies have conducted motor mapping during active contraction of target muscles (Byrnes et al., 1999; Classen et al., 1998; Marconi et al., 2007; Ngomo et al., 2012, 2015; Reilly & Mercier, 2008; van de Ruit & Grey, 2016; Wassermann et al., 1993; Wilson et al., 1993, 1995). Of those, only five (to our knowledge) have compared motor mapping measures obtained at rest and during active muscle contraction with contradictory results (Classen et al., 1998; Marconi et al., 2007; Ngomo et al., 2012; van de Ruit & Grey, 2016; Wilson et al., 1995). Wilson et al. (1995) found the active map center of gravity (CoG) and hotspot (location with highest peak‐to‐peak MEP amplitude) to be more medial than at rest, but otherwise all other map parameters were similar (Wilson et al., 1995). In contrast, this shift was not observed in later studies using similar paradigms (Classen et al., 1998; Ngomo et al., 2012). In fact, Ngomo et al. (2012) showed no differences between their resting and motor maps (Ngomo et al., 2012). Meanwhile, Marconi et al. (2007) and van de Ruit and Grey (2016) reported that, compared to resting maps, active maps had increased map area and volume, which they assumed was due to scaling of the map with voluntary contraction (Marconi et al., 2007; van de Ruit & Grey, 2016). Small sample sizes (*N* ≤ 8) limit the ability to draw strong inferences from many of these studies (Classen et al., 1998; Marconi et al., 2007; Wilson et al., 1995), however, a possible contributor to the inconsistent findings described were differences in mapping methods beyond the motor state of the target muscle.

Variations in stimulation paradigms (grid size, grid spacing, total stimulation number, stimulation intensity), target muscles, level of voluntary muscle activation (% maximum voluntary contraction), and targeting methods (pre‐defined grid, pseudo‐random, neuro‐navigation) may influence motor map measurements. Grid size and spacing pre‐determine the stimulation sites that are used to create motor maps. The small grids and fewer total stimulations used by previous studies may have led to incomplete maps while larger distances between stimulation sites may result in lower resolution. Since map area and volume increase with stimulation intensity (van de Ruit & Grey, 2016), the mapping stimulation intensity used is also important, especially when comparing these measures. Despite it being well‐established that minimum stimulation thresholds are different in hand muscles during active contraction compared to at‐rest (Buccolieri et al., 2004; Christova et al., 2006; Darling et al., 2006), many previous studies used the same stimulation intensity for both resting and active maps (Classen et al., 1998; Marconi et al., 2007; van de Ruit & Grey, 2016; Wilson et al., 1995). Furthermore, although there is evidence that voluntary muscle activation greater than 10% of MVC has little effect on MEP amplitude for hand muscles (Helmers et al., 1989; Taylor et al., 1997), van de Ruit & Grey, 2016 found increasing contraction led to a corresponding increase in motor map area and volume (van de Ruit & Grey, 2016). Co‐contraction of other muscles may increase MEP amplitude and in turn influence map parameters (Tazoe & Perez, 2021). Isolation of the target muscle may therefore be important to limit the confounding effects of additional hand muscle co‐activation on active motor maps (Izumi et al., 2000). Both the orientation of the coil and the selection of which targets to stimulate can also affect TMS parameters (D'Ostilio et al., 2016; Gomez‐Tames et al., 2018; Pascual‐Leone et al., 1994; Sakai et al., 1997). To overcome the human error that may arise with traditional manual TMS (Sparing et al., 2008), TMS robot technologies combined with individualized neuro‐navigation to ensure correct coil orientation have been developed to increase precision (Grab et al., 2018). The use of these novel TMS mapping techniques may improve motor map precision and potentially facilitate comparisons of rest and active motor maps, but no such studies have been reported.

It is not clear what the relationship between active and resting maps are because methodological choices limit interpretability. Therefore, there is a need for further comparison between active and resting motor maps, using a large grid with small spacing, controlled and isolated contraction of the target muscle, with stimulation intensities specific to the motor thresholds obtained under each condition (rest or active), combined with precisely controlled targeting, and motor performance measures. The present study utilized personalized robot‐controlled, image‐guided neuro‐navigation TMS techniques to compare motor map measures during active muscle contraction and at rest. We hypothesized that active motor maps would have larger area and volume compared to those collected at rest and stronger positive correlations with motor performance.

## 2 MATERIALS AND METHODS

### 2.1 Participants

Twenty healthy right‐handed participants (8 female) aged 19–38 years (mean age 25.7 ± 5.0 years) participated in the study. Handedness was assessed using the Modified Edinburgh Handedness Inventory (Oldfield, 1971); those that had a score of 60 but identified as right‐handed were included in the study. Participants had received a structural magnetic resonance imaging (MRI) within 2 years of this study. Participants were pre‐screened for any contraindications to non‐invasive brain stimulation or MRI (metallic implants, pregnancy). Participants were instructed to refrain from caffeine consumption at least 2 h before the session and from any physically strenuous upper limb activities at least 24 hours before the session. Participants provided informed consent before participating. Procedures were approved by the local Conjoint Health Research Ethics Board at the University of Calgary (REB 17–2225) and in accordance with the Tri‐Council Policy Statement 2 (TCPS2). All procedures occurred at the Alberta Children's Hospital Pediatric Non‐Invasive Brain Stimulation Laboratory which has safely administered more than 3.5 million stimulations to >300 participants (Zewdie et al., 2020).

### 2.2 Experimental design

**FIGURE 1:**
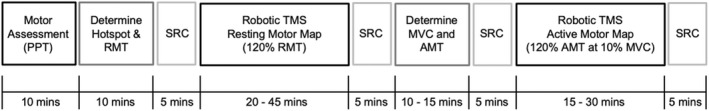
Study design. Outline and breakdown of participant progression through the experiment day is demonstrated. AMT, Active motor threshold; MVC, maximum voluntary contraction; PPT, purdue pegboard test; RMT, resting motor threshold; SRC, stimulus response curve.

Participants attended the lab for one experimental session, divided into two testing blocks (resting and active mapping) (see Figure 1). Participants completed functional motor assessments, neuro‐navigated robotic TMS brain stimulation, and tolerability forms. A 5 to 15‐min rest (optional bathroom or water break) was provided between the resting and active mapping procedures as needed.

### 2.3 Measures

#### 2.3.1 TMS procedures

**FIGURE 2:**
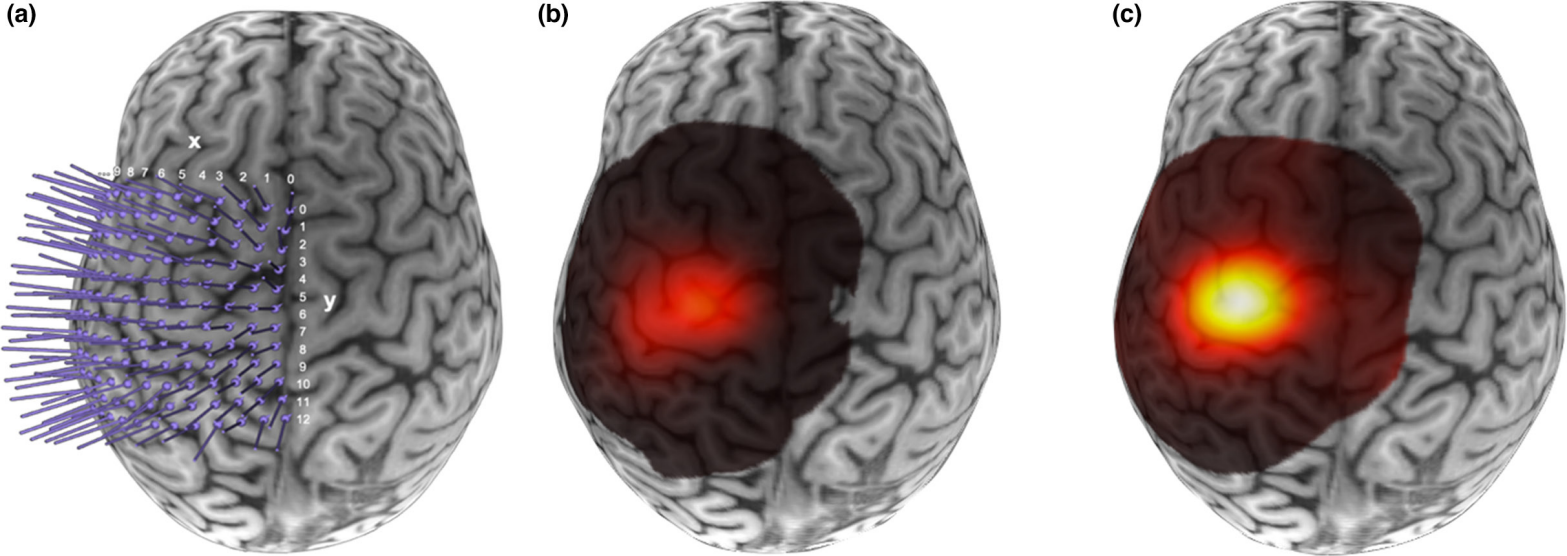
Sample resting and active motor maps. Examples of a participant (a) 12 × 12 grid (0.7 cm spacing) for neuro‐navigated robotic transcranial magnetic stimulation, and their (b) resting motor map and (c) active motor map overlaid on their 3D brain surface. The grid is centered around the anatomical ‘hand knob’; typically positioned around 4,5 (*x*,*y*).

Prior to the study session, each participant's T1 MRI scan was rendered into a 3D brain model using neuro‐navigation software (Brainsight2, Rogue Research). A 12 × 12 rectangular coordinate grid with 7 mm spacing was placed on the 3D brain surface, centered over the anatomical ‘hand knob’ (Caulo et al., 2007; Yousry et al., 1997) in the left hemisphere with the right edge of the grid overlaid parallel over the interhemispheric fissure, and curved over the individualized surface of the participant's MR brain reconstruction (Figure 2a) (Giuffre et al., 2019). The anatomical hand knob is approximately centered around grid coordinates 4,5 (*x*,*y*). Grid trajectories were oriented so the coil position was at a 45° angle to the midline with the handle pointing backwards to preferentially induce current in the posterior‐to‐anterior direction in the cortex. The coil angle (tilt and rotation) was adjusted between the stimulation locations to take anatomy into account. All TMS procedures were conducted using a 70 mm figure‐eight Airfilm coil with a Magstim Rapid Stimulator (Magstim) attached to a robotic TMS system (Axilum Robotics) with participants seated comfortably in the TMS robot chair with their forearm supported. Using infrared passive sensors, an optical tracking system (Polaris, NDI Medical Solutions), and the neuro‐navigation software (Brainsight2), the participant's head, TMS coil, and TMS robot were co‐registered in three‐dimensional space, allowing the TMS robot to remain precisely aligned on the target trajectories including near‐real‐time motion correction.

Electromyographic (EMG) activity was recorded from the right first dorsal interosseous (FDI) muscle using silver/silver‐chloride (Ag/AgCl) surface electrodes with the active electrode placed over the muscle belly and the reference electrode placed over the metacarpophalangeal joint of the index finger. EMG signal was amplified by 1000, band‐pass filtered from 20 to 2000 Hz, and digitized at 5000 Hz with CED 1401 hardware and Signal 6.0 software (Cambridge Electronic Design). EMG signals were monitored online for movement‐related activity on Signal. TMS‐induced muscle activations were recorded as motor evoked potentials (MEPs).

In order to determine optimal mapping TMS intensity, the ‘hotspot’ for the contralateral FDI was determined by stimulating grid targets positioned around the anatomical ‘hand knob’ until the optimal scalp grid point for evoking the largest MEP amplitude was established. Resting motor threshold (RMT), defined as the minimum stimulus output required to evoke a MEP of at least 50 μV in at least 5 out of 10 successive stimuli, was then established (Rossini et al., 1994). For the resting motor mapping block, participants were permitted to watch a (G or PG rated) movie that they deemed engaging, but not overly exciting/thrilling to balance the attentional focus between the two conditions (resting and active).

For the active block, the right hand was placed in a custom‐built device that held the hand so that the participant could perform an isometric contraction of their right FDI with limited activation of the other hand and arm muscles. Maximum voluntary contraction (MVC) was determined by having the participant maximally isometrically abduct their index finger for approximately 5 s. This process was repeated four times, and the largest rectified EMG signal was recorded as the MVC. The participant observed their live trace of a smoothed (200 ms envelope) and rectified EMG biofeedback on a large TV screen. During active TMS trials, a target horizontal line at 10% MVC was also presented. Active motor threshold (AMT) was determined as the minimum stimulus output required to elicit a MEP with a peak‐to‐peak amplitude greater than background EMG in ≥5/10 trials while contracting to 10% MVC.

#### 2.3.2 Motor mapping (resting and active)

Resting motor mapping was performed by delivering TMS pulses at 120% of FDI RMT. For each grid target, four consecutive stimulations with a 1 s interstimulus interval were delivered (stimulus onset at 0.5 s). A point was judged in real‐time and later confirmed offline by a trained TMS expert as either responsive (≥2/4 stimulations [Giuffre et al., 2021; Grab et al., 2018] evoked a >50 μV MEP) or non‐responsive (failure to do so). A motor map was generated as previously described where targets were stimulated sequentially starting from the hotspot, moving up and down the grid rows until a non‐responsive point was reached (Giuffre et al., 2019; Grab et al., 2018). The map was complete when all responsive sites were fully encircled (including diagonal grid points) by non‐responsive sites. Trials with excessive background EMG activity were excluded and recollected.

Active motor mapping was performed in a similar fashion. Stimuli at 120% of FDI AMT were delivered while the participant contracted to 10% of their MVC for four stimulations (1.5 s interstimulus interval, stimulus onset 1.5 s). Participants were instructed to relax their FDI while the coil was moving to the next grid point. Each point was judged as responsive or not as above with a MEP amplitude cut‐off of peak‐to‐peak > rectified EMG amplitude during contraction of 10% MVC. Trials where contraction was not at 10% MVC, were excluded and recollected. These methods have been shown to provide good to excellent reliability for motor map parameters (Kahl, Giuffre, et al., 2021).

#### 2.3.3 Stimulus response curve

To ensure that differences in resting and active map measures were not due to a change in excitability over time, immediately prior to and following each motor map (resting and active), a stimulus‐response curve (SRC) was derived at the hotspot determined prior to mapping. Starting from 100% TMS stimulus output intensity and decreasing in intervals of 5%, four stimulations were delivered until the intensity at least 20% less than the participant's RMT or AMT was reached. If stimulations at 100% were not tolerable, SRC stimulations started at 120% of the participant's RMT or AMT rather than 100% of the output intensity.

### 2.4 Creating the map

Data frames were extracted from an exported Signal file and analyzed offline using a custom R script (R Core Development Team, 2015). The primary outcomes were differences in motor map area and volume between resting and active conditions. Secondary mapping outcomes included center of gravity (CoG) location (*x* and *y* coordinates), hotspot location (*x* and *y* locations), hotspot displacement (Euclidian distance between hotspot and CoG locations), hotspot magnitude (Grab et al., 2018) and map aspect ratio (van de Ruit & Grey, 2016). Map area was calculated as the grid spacing (7 × 7 mm) multiplied by the total number of responsive sites. Map volume was calculated as the cumulative sum of grid spacing (2D area) multiplied by the mean MEP amplitude at each responsive site. CoG was calculated in the same manner as Wassermann et al. (1992), weighing the motor representation of each coordinate point (see equation) (Wassermann et al., 1992).

$$x_{\mathrm{COG}}=\frac{\sum x_{i}M_{i}}{\sum M_{i}},y_{\mathrm{COG}}=\frac{\sum y_{i}M_{i}}{\sum M_{i}}$$

where *x*_*i*_ = *x* grid coordinate, *y*_*i*_ = *y* grid coordinate, *M*_*i*_ = the corresponding grid point's mean MEP peak‐to‐peak amplitude.

To quantify the shape of the map we quantified the map aspect ratio, the ratio of the major and minor axes of an ellipse fitted around the responsive sites, which describes the shape of the excitable area (van de Ruit & Grey, 2016). Similarly to van de Ruit and Grey (2016), for active maps, approximately 15% (±5%) of hotspot magnitude was used as a cut‐off so that the ellipse border would not fall outside the border of the map.

#### 2.4.1 Assessing motor performance and TMS tolerability

Dominant (right) hand motor function was quantified using the Purdue Pegboard Test (PPT) (Tiffin & Asher, 1948). While the PPT consists of four unimanual and bimanual subtests, only the unimanual right‐hand peg placement subtest was used in this study. In this test, participants used their right hand to precisely place as many small, rounded pegs as possible in 30 seconds into a row of holes. This test was repeated 3 times and the average score was recorded.

A modified brain stimulation safety and tolerability questionnaire (Garvey et al., 2001; Zewdie et al., 2020) was administered following all TMS procedures. This questionnaire captures any adverse events and the duration and severity of any symptoms reported.

### 2.5 Statistical analysis

All data are reported as mean ± standard deviation (SD) unless otherwise stated. Normality was assessed using the Shapiro–Wilk's test. Motor map parameters: area (mm^2^), volume (mm^2^ × mV), CoG coordinates, hotspot coordinates, hotspot magnitude (mV), map aspect ratio (a. u.), and motor thresholds (% maximum stimulator output; MSO) were compared between resting and active maps using paired samples Student's *t*‐test. Cohen's d (*d*) for paired measures was used as an estimate of effect size. For measures that were not normally distributed, the Wilcoxon signed‐rank test was used (with the rank biserial correlation, *r*, used as an estimate of the effect size). Bland–Altman plots were used to measure the agreement between resting and active for each of the map parameters and for motor thresholds (Martin Bland & Altman, 1986). In these plots, each data point represents the difference between two measurements plotted against the mean of the two measurements; with the mean value of difference, lower limit of agreement (LOA‐L), and upper limit of agreement (LOA‐U) shown. For each SRC, a sigmoidal (Boltzman) curve was fitted in GraphPad (Version 9.2.0, GraphPad Prism, San Diego, USA) and the slope was calculated. SRC slope was compared pre‐ and post‐ each map (resting and active) to examine whether global measures of corticospinal excitability changed throughout the experimental session. The Euclidian distances between resting and active hotspots and CoGs were calculated between *x* and *y* grid coordinates and then multiplied by 7 mm (grid spacing) to describe the variation in these positions. For the *t*‐tests and correlation coefficients, the *p* value was adjusted for multiple comparisons using the Holm‐Bonferroni method. The threshold for rejecting the null hypothesis was *p* < 0.05. The Bayes Factor (BF_10_, Cauchy prior = 0.707) was calculated to give the likelihood of the data under both the null and alternative hypotheses, with BF_10_ > 3 or <0.3 considered as evidence for the alternative or null hypotheses respectively. All statistical tests were performed in R (R Core Development Team, 2015) using Jamovi (Version 1.1.9.0) (jamovi, 2020).

## 3 RESULTS

### 3.1 Population

Table 1 shows the participant demographics. All 20 participants completed the study with no serious adverse events.

**TABLE 1:** Participants

ID	Age (years)	Sex	EH	RMT (% MSO)	AMT (% MSO)	MVC (mV)	PPT
1	23.5	M	80	61	58	0.89	15.3
2	28.6	M	100	48	49	1.04	16.3
3	25.8	F	80	54	52	1.00	18.3
4	32.1	M	90	52	44	0.99	15.7
5	20.0	F	60	54	44	1.30	19.3
6	20.1	F	80	66	62	1.11	16.7
7	20.2	F	100	42	40	0.73	16.7
8	25.7	M	70	55	55	1.02	15.7
9	32.1	M	80	53	46	1.21	14.7
10	28.5	M	90	50	53	0.83	16.7
11	30.9	M	80	50	47	1.09	16.7
12	25.1	M	100	61	60	1.01	14.3
13	20.6	M	60	60	51	1.31	14.7
14	37.6	F	100	74	64	0.66	15.0
15	23.8	M	80	59	51	1.33	16.0
16	27.7	M	90	48	39	0.93	14.7
17	24.0	F	100	56	49	0.83	13.7
18	19.3	F	80	63	58	1.26	13.0
19	20.5	F	95	55	57	0.44	15.7
20	28.5	M	80	53	47	0.48	15.3

Abbreviations: AMT, active motor threshold (% maximal stimulator output); EH, Edinburgh handedness score; MSO, maximum stimulator output; MVC, maximum voluntary contraction (mV); PPT, purdue pegboard test score (mean number of pegs); RMT, resting motor threshold (% maximal stimulator output).

Group descriptive statistics for all map parameters (area, volume, CoG coordinates, hotspot coordinates, hotspot magnitude) and motor threshold (RMT and AMT) for resting and active maps are presented in Table 2.

**TABLE 2:** Summary of comparison between resting and active TMS map measures

	Resting mean ± SD/median [IQR]	Active Mean ± SD/median [IQR]	Comparison resting versus active		Association between resting and active	
			*T*	*p*	Rho	*p*
Area (mm^2^)	889 ± 294	4403 ± 669	23.5	0.010*	0.3	0.988
Volume (mm^2^ × mV)	408 ± 277	5461 ± 1774	12.7	0.010*	0.1	1.000
CoGx (a.u.)	4.52 ± 0.78	4.54 ± 0.71	0.3	0.734	0.9	0.009^†^
CoGy (a.u.)	5.28 ± 1.33	5.34 ± 1.10	0.5	0.622	0.9	0.009^†^
Hotspotx (a.u.)	4.15 ± 1.14	4.45 ± 0.89	1.5 (*W*)	0.996	0.6	0.025^†^
Hotspoty (a.u.)	5.05 ± 1.39	5.30 ± 1.49	1.0 (*W*)	1.000	0.7	0.009^†^
Hotspot Mag (mV)	1.34 ± 0.77	6.34 ± 1.77	12.0	0.010*	0.2	1.000
Hotspot Disp (mm)	0.83 ± 0.44	0.85 ± 0.48	0.1	1.000	−0.2	1.000
Aspect ratio (a.u.)	1.6 [1.4–1.8]	1.7 [1.6–1.8]	81 (*W*)	1.000	0.2	1.000
Motor Threshold (% MSO)	56.00 ± 7.54	49.50 ± 6.95	5.7	0.010*	0.8	0.009^†^

Abbreviations: Aspect ratio, ratio of the major and minor axes of an ellipse fitted around the responsive sites; a.u., arbitrary unit; CoGx, center of gravity *x* coordinate; CoGy, center of gravity *y* coordinate; Hotspot Disp, hhotspot displacement (Euclidian distance between hotspot and CoG locations); hostpot Mag, hotspot magnitude; Hotspotx, hotspot *x* coordinate; Hotspoty, hotspot *y* coordinate; MSO, maximum stimulator output.

* Difference in measure between active and resting motor map (*p* < 0.05, Holm‐Bonferroni adjusted).

^†^ Association in measure between active and resting motor map (*p* < 0.05, Holm‐Bonferroni adjusted).

### 3.2 Comparing resting to active TMS motor map measures

**FIGURE 3:**
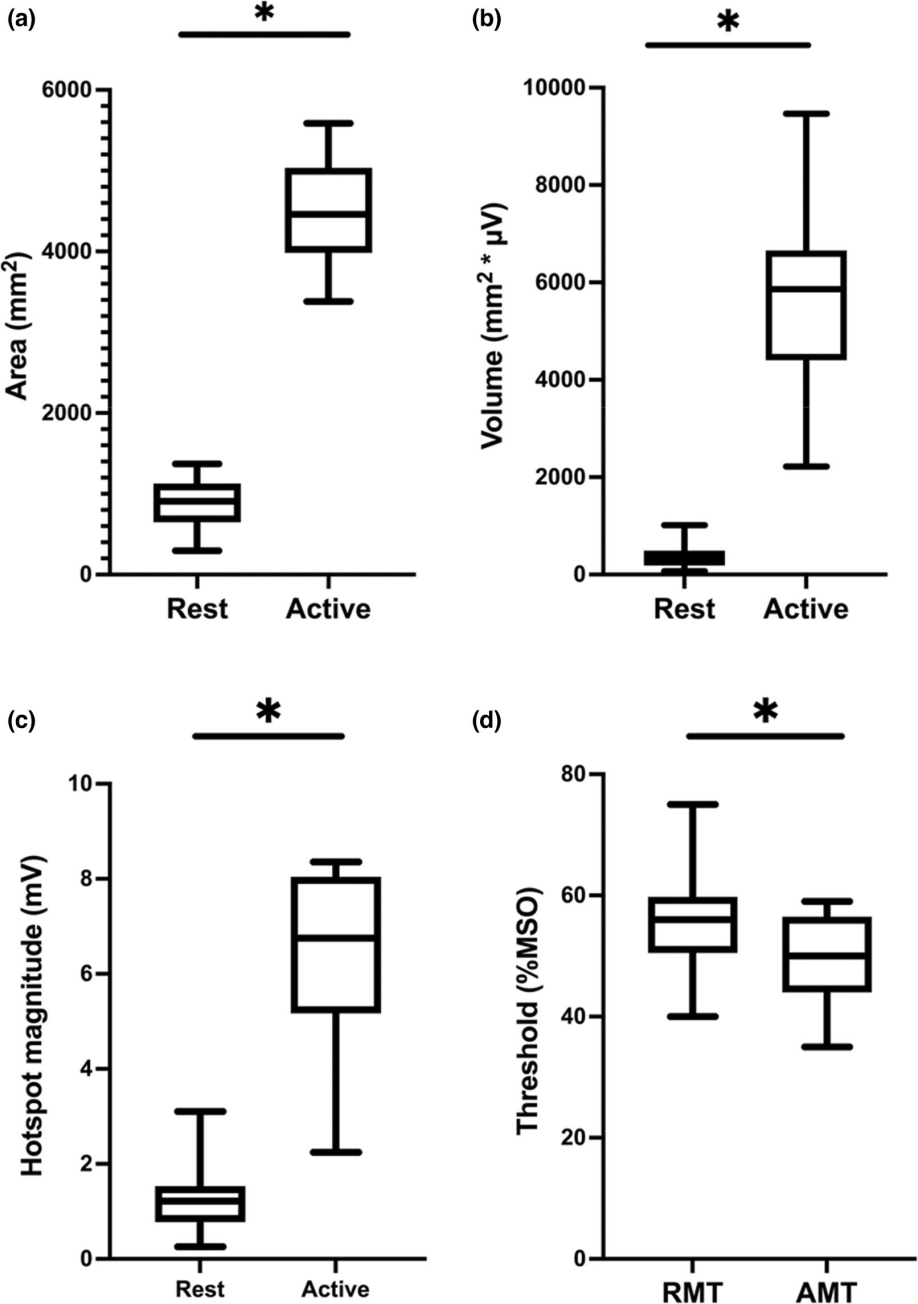
Differences in TMS motor map measures. Pairwise comparisons for differences in (a) area, (b) volume, (c) hotspot magnitude, and (d) motor threshold between resting and active motor maps. * Significantly different (*p* < 0.05).

Map area (*T*_[19]_ = 23.5, *p* < 0.001, *d* = 5.2, BF_10_ = 2.7e + 12) and volume (*T*_[19]_ = 12.7, *p* < 0.001, *d* = 2.8, BF_10_ > 1000) were larger for the active maps compared to the resting maps. Hotspot magnitude (*T*_[19]_ = 12.0, *p* < 0.001, *d* = 2.7, BF_10_ > 1000) and motor thresholds (*T*_[19]_ = 5.7, *p* < 0.001, *d* = 5.8, BF_10_ > 1000) were also larger for the active maps compared to the resting maps (Figures 2b,c and 3). No significant differences were observed between states for the remaining map outcome measures: CoGx (*T*_[19]_ = 0.3, *p* = 0.734, *d* = 0.1, BF_10_ = 0.2), CoGy (*T*_[19]_ = 0.5, *p* = 0.622, *d* = 0.1, BF_10_ = 0.3), hotspotx (*W = 22*, *p* = 0.996, *r* = 0.3, BF_10_ = 0.6), hotspoty (*W* = 27.5, *p* = 1.000, *r* < 0.1, BF_10_ = 0.4), hotspot displacement (*T*_[19]_ = 0.1, *p* = 0.892, *d* = 0.0, BF_10_ = 0.2), and aspect ratio (*W* = 81, *p* = 1.000, *r* = 0.2) (Table 2). The averaged Euclidian distance between resting and active hotspots and CoGs were 8.47 and 3.78 mm respectively. For resting motor mapping, an average of 23 sites required resampling per map, while for active mapping only an average of three sites needed to be resampled per map.

SRC slope did not change following resting (Pre: 0.10 ± 0.07, Post: 0.11 ± 0.07; *p* = 0.448) or active mapping (Pre: 0.19 ± 0.12, Post: 0.15 ± 0.10; *p* = 0.164).

### 3.3 Agreement between resting and active TMS motor map measures

#### 3.3.1 | Bland–Altman

**FIGURE 4:**
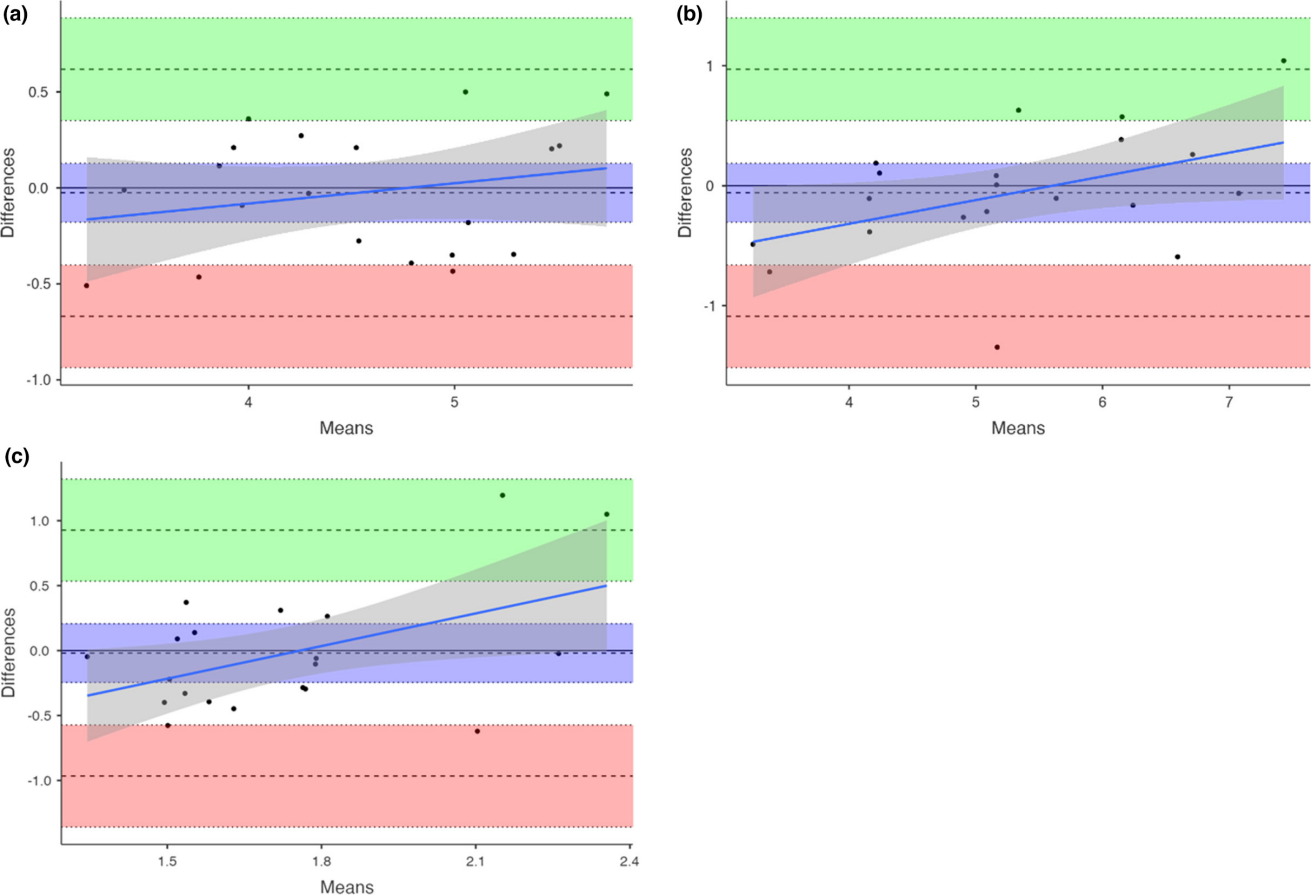
Agreement between resting and active TMS motor map measures. Bland–Altman plots of (a) Center of Gravity *x* coordinate, (b) Center of Gravity *y* coordinate, (c) aspect ratio. These plots show the agreement between data points for resting and active motor mapping outcomes in relation to the mean of the two values. Values falling along the line at *y* = 0 show strong agreement. Data points within ±2 standard deviations are indicated by the dashed lines above and below. The green and red shading represent the upper and lower 95% confidence intervals. The blue line indicates the proportional bias line and the gray shading represents the proportional bias line 95% confidence intervals.

Bland–Altman plots showed excellent agreement between resting and active CoGx (LOA‐L = ‐0.722 [95% Confidence Interval (CI) = −1.0, −0.4] and LOA‐U = 0.620 [0.3, 0.9]) and CoGy (LOA‐L = ‐1.144 [−1.6, −0.7] and LOA‐U = 0.976 [0.5, 1.4]), and aspect ratio (LOA‐L = ‐0.966 [−1.4, −0.6] and LOA‐U = 0.927 [0.5, 1.3]). However, poor agreement was found between most other measures (Figure 4, Table 3).

**TABLE 3:** Agreement between resting and active TMS motor map measures

Measure	Mean	LOA‐L	95% CI		LOA‐U	95% CI	
			Lower	Upper		Lower	Upper
CoGx	4.53	−0.67	−0.9	−0.4	0.62	0.4	0.9
CoGy	5.31	−1.09	−1.5	−0.7	0.97	0.5	1.4
Area	2646	−4826	−5372	−4281	−2200	−2745	−1655
Volume	2934	−8535	−9980	−7090	−1572	−3017	−127
Hotspot x	4.30	−2.11	−2.9	−1.4	1.51	0.8	2.3
Hotspot y	5.18	−2.35	−3.2	−1.5	1.85	1.0	2.7
Hotspot Mag	3.84	−8.66	−10.2	−7.1	−1.35	−2.9	0.2
Hotspot Disp	0.84	−1.38	−1.9	−0.8	1.34	0.8	1.9
Aspect Ratio	1.74	−0.97	−1.4	−0.6	0.93	0.5	1.3
Motor Threshold	53.50	−3.63	−7.0	−0.3	12.43	9.1	15.8

Abbreviations: Aspect Ratio, ratio of the major and minor axes of an ellipse fitted around the responsive sites; CoGx, center of gravity *x* coordinate; CoGy, center of gravity *y* coordinate; Hotspot disp, hotspot displacement (Euclidian distance between hotspot and center of gravity locations); Hotspot Mag, hotspot magnitude (mV); Hotspot *x*, hotspot *x* coordinate; Hotspot *y*, hotspot *y* coordinate; LOA‐L, lower limit of agreement; LOA‐U, upper limit of agreement.

#### 3.3.1 Correlations between maps

There were significant correlations between active and resting maps for CoGx (rho = 0.908, *p* = 0.010), CoGy (*rho* = 0.924, *p* < 0.001), hotspotx (rho = 0.601, *p* = 0.030), hotspoty (*rho* = 0.730, *p* = 0.010), and motor threshold (rho = 0.814, *p* = 0.010). All other measures did not demonstrate an association between resting and active conditions (Table 2).

There were no significant associations between PPT score and motor map parameters (all *p* > 0.05).

### 3.4 Safety and tolerability

**FIGURE 5:**
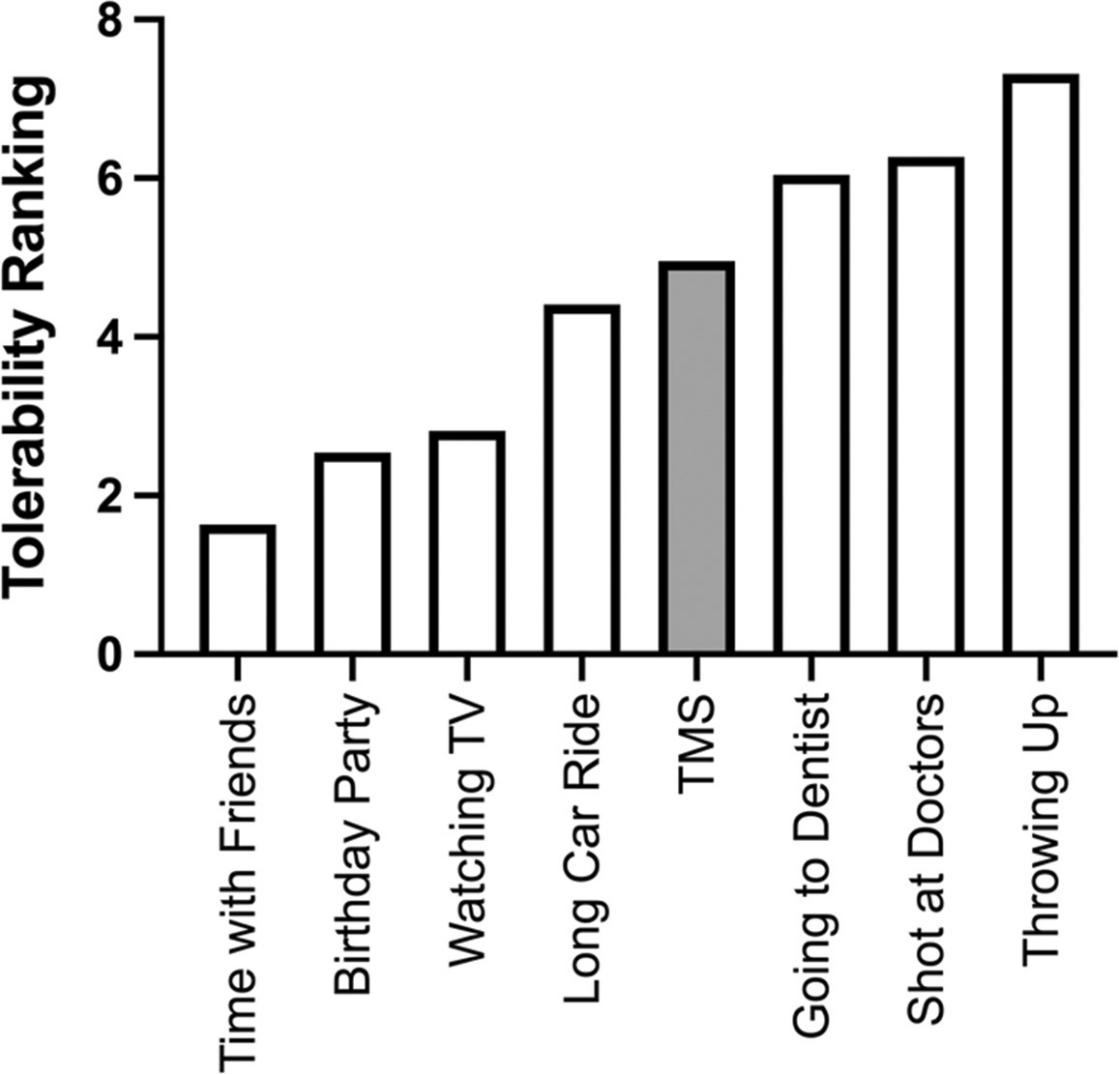
Robotic TMS motor mapping tolerability. Tolerability rankings of TMS motor mapping (in gray). Participants were instructed to rank the eight activities from favorite (1) to least favorite (8) using each number once.

All procedures were well tolerated. The mean rank order preference for robotic TMS motor mapping was 5th favorite; not as good as time with friends, a birthday party, watching TV, or a long car ride, and better than going to the dentist, a shot/injection at the doctor's, and throwing up (Figure 5).

## 4 DISCUSSION

The aim of the present study was to compare TMS motor maps recorded with the muscle at rest and during active contraction. Using robot‐guided neuro‐navigated TMS, we found that map area, volume, and hotspot magnitude were all higher in active maps compared to resting. In contrast, both map centrality (CoG and hotspot location) and map shape (aspect ratio) were similar in both types of mapping. Outcomes from either map type were not strongly associated with a simple measure of hand motor function. These data add to the existing results showing large differences in map size but not map centroid or shape and indicate maps scale with voluntary contraction. It is possible that active maps may reflect functionally different cortical representations of the target muscle compared to resting maps.

The magnitude of the differences in motor map area and volume seen in the present study are similar to those previously reported by van de Ruit and Grey (2016) who showed that map size scales linearly with muscle contraction intensity (van de Ruit & Grey, 2016). The likely cause of these differences are the effects of active muscle contraction on the excitability of the motor cortex and motoneuron pool, which leads to increased size and number of corticospinal volleys evoked by TMS (Di Lazzaro et al., 1998). TMS of the primary motor cortex evokes several volleys of corticospinal activity, of which the earliest waves are thought to originate from direct axonal activation of pyramidal tract neurons (D‐waves). Later waves are thought to originate from indirect, trans‐synaptic activation of mono‐and polysynaptic inputs to the pyramidal tract neurons (I‐waves) (Di Lazzaro & Ziemann, 2013; Volz et al., 2015). In the contracted state, the summation of descending voluntary impulses from cortical areas, afferent impulses from muscle spindles, and descending potentials secondary to the TMS alter corticospinal summation mechanisms (Claus et al., 1988; Rossini et al., 1987a, 1987b; Sarfeld et al., 2012). This results in increased amplitude, decreased threshold, and shortened latency of the TMS‐evoked muscle response (Kaneko et al., 1996). The increased amplitude of the muscle response, in turn, results in larger MEPs at the stimulation sites of a motor map and the decreased threshold in a larger area (number of stimulation sites) in response to TMS. While TMS stimulation at rest rarely elicits D‐waves, they have been produced at higher stimulation outputs (Kaneko et al., 1996). TMS delivered at 120% AMT may elicit D‐waves induced by direct activation of the axon hillock, although this is considered unlikely (Di Lazzaro et al., 1998; Di Lazzaro & Ziemann, 2013; van de Ruit & Grey, 2016). A more likely explanation is that there are a great number of larger I‐waves induced by voluntary contraction (Di Lazzaro et al., 1998). As a result, as seen in the present study, active motor maps are larger in area, volume, and hotspot magnitude compared to resting motor maps.

It is believed that voluntary contraction may reflect the excitability of cortical neurons in a more functionally‐relevant way compared to a resting condition (Devanne et al., 1997). The larger size of the active motor maps seen here suggests that active maps may reflect different cortical representations of the target muscle compared to resting maps. Because resting motor maps are smaller than active maps, it is possible that they only capture a fraction of the functionally discrete cortical region involved in motor performance. For example, in participants with spinal cord injuries, TMS motor maps obtained at rest were larger than those of age‐matched healthy controls, yet smaller during voluntary FDI activation. Furthermore, of the participants with spinal cord injuries, those that had larger sensory deficits had larger reductions in active motor map area (Tazoe & Perez, 2021). For cases where TMS motor mapping is used to delineate the cortex involved in motor control to preserve prior to surgically removing brain tissue, active motor mapping may provide a more functionally‐relevant outline of the region. For studies using TMS motor maps to probe differences or changes in cortical organization between clinical populations or after an intervention, active maps may be able to detect underlying differences that are outside of the resting map region (Kahl, Kirton, et al., 2021). For example, a small focal stroke may lie in a location not captured by resting motor mapping, but its influence on hand function may be revealed by an active map.

Although motor map area, volume, and magnitude scaled in size with voluntary contraction, the CoG, map aspect ratio, and hotspot locations were not different between resting and active motor maps. van de Ruit and Grey (2016) suggested that cortical neurons along the perimeter of the target muscle's cortical representation are not equally excitable in the two mapping methods (van de Ruit & Grey, 2016). Therefore, since the center of the map and the shape remain constant between different types of motor map acquisition protocols, the uniform expansion of map borders is the defining difference between active and resting motor mapping.

To our knowledge, no study has compared resting and active motor maps collected with a TMS robot. This novel method may provide increased reproducibility (Giuffre et al., 2021; Kahl, Giuffre, et al., 2021) and accuracy while reducing the time required for mapping procedures (Grab et al., 2018). In previous studies, neuro‐navigation protocols have varied drastically, from a cap with pre‐marked sites (Wilson et al., 1995) or a grid fixed on the subject's head (Marconi et al., 2007), to a frameless stereotaxic neuro‐navigation system (Ngomo et al., 2012; van de Ruit & Grey, 2016). While some studies outline coil orientation methods, none report how coil orientation was controlled and maintained throughout the mapping procedure. The robotic TMS paired with nteuro‐navigation used in the present study ensured that the specific orientation of the TMS coil was maintained in 3D space with near real‐time head motion correction. Motor map grid size, spacing, and stimulation protocols may have impacted outcome measure reproducibility as well. Previous studies mentioned small grid size (e.g., 6 × 6 or 7 × 7) as a limitation as they were unable to collect complete maps (Marconi et al., 2007; van de Ruit & Grey, 2016). Additionally, the 2‐min mapping technique used by van de Ruit and Grey (2016), while efficient, may sacrifice map resolution because the spacing between stimulation sites is inconsistent and may leave gaps (van de Ruit & Grey, 2016). The present study used a much larger grid (12 × 12) to prevent such data loss. Previous studies have a number of limitations, which we addressed in the present study.

To ensure the differences found in resting and active mapping measures were not due to changes in cortical excitability as a result of the mapping itself, this study collected stimulus‐response curves (SRC) before and after each map. These curves are believed to provide information about the neurophysiological strength of intracortical and corticospinal connections and the thresholds of the neuron populations being stimulated (Chen, 2000; Devanne et al., 1997; Hallett, 2000; Ridding & Rothwell, 1997). The resulting SRC curves showed there were no changes in cortical excitability after each map. Additionally, to prevent muscle fatigue impacting cortical excitability and thus motor map outcomes, resting motor mapping was conducted prior to active motor mapping.

As with many TMS measures, one limitation of the present study is the inability to control for upstream effects on cortical motor maps. Differences in visual input, attentional state, and sensory involvement during motor mapping may have influenced corticospinal excitability. It has been established that action‐related visual information can modulate the connectivity to the motor system. Specifically, action‐related instructions or visual signals increase the excitability of facilitatory interactions between the dorsolateral prefrontal cortex and muscle representations in the primary motor area (Hasan et al., 2013). There may have also been increased ascending volleys of sensory input during active mapping that could have contributed to differences in map measures. While active motor mapping may require more attention for the participant to maintain accurate contraction, the present study found resting mapping to require a greater amount of resampling due to participants inability to fully relax their FDI, requiring the participant to actively relax. Therefore, though not in equivalent level, maintaining a relaxed muscle also requires a great level of attention. As such, both intentional contraction and total relaxation of a target muscle may pose as difficult tasks for neurologically impaired participants and the difficulty of holding the 10% FDI contraction might be amplified in manual motor mapping. Future research should consider exploring various tasks with differing levels of concentration and attention requirements, such as completing visual tasks or maintaining stable leg muscle EMG, to see how these affect the two different types of motor maps. Another study design limitation is in the lack of randomization with all participants undergoing the same order of mapping: Resting first, followed by active. Although SRCs were similar after both maps, fatigue, boredom, or other issues may have affected mapping outcomes. Therefore, future studies comparing these two mapping methods should randomize participants with regards to which participants received active or resting mapping first to make the study more rigorous. Furthermore, SRC was collected only at one grid point—the pre‐determined map hotspot—therefore, change in excitability before and after mapping was only measured for one location. Measuring SRC at every responsive grid location and finding a similar consistency in curves before and after maps would allow the present lack of change in excitability to be generalized.

One observation that can be anecdotally drawn from the present study is that current motor map measures (i.e., area, volume, etc.) are potentially limited in their ability to describe motor maps because they lack the ability to characterize the shape of the map. Visual inspection of resting and active motor maps (Figure S1) suggests profound differences in map geometry which may not be adequately described using traditional map parameters. Future explorations of additional motor map quantification measures are required to better detect differences and changes in motor maps. While resting motor map reliability has been fairly well established (Giuffre et al., 2021; Malcolm et al., 2006; Mortifee et al., 1994), there is currently only one study (to our knowledge) that has investigated active motor map reliability across time. Future studies using robotic TMS motor mapping are required to better understand the neurophysiology underlying active motor maps and their reliability.

PPT scores did not correlate with motor map measures. This is consistent with previous findings that cortical excitability, measured as TMS‐induced MEP facilitation, was not correlated with PPT performance (Brouwer et al., 2001). A possible reason for this may be that the PPT performance is not solely dependent on the FDI and thus, may not be a specific enough tool to measure this specific muscle. The proficient grasping of the small pegs entails simultaneous motion at multiple joints, involving multiple upper limb muscles (Gonzalez et al., 2017). PPT scores may largely be affected by thumb and index grasp, arm, wrist, and shoulder skills, rather than index finger abduction; thus, PPT may correlate with maps of these muscles instead. The present study also did not control for motor history of the FDI muscle, such as years of playing a musical instrument involving high dexterity and acuity of FDI movement. It would be interesting to see whether motor map measures correlate with musical skills (e.g., playing the piano) or looking at differences between trained muscles (e.g., musicians versus non‐musicians). The lack of correlation between PPT and motor maps could also be due to the established learning and practice effects with PPT (Noguchi et al., 2006). Therefore, future studies should investigate whether learning modifies this relationship.

## 5 CONCLUSIONS

Although previous studies comparing TMS motor maps obtained at rest and during voluntary contraction exist, the present study adds novel robotic methodology to explore this comparison, utilizing robotic TMS technology paired with personalized neuro‐navigation. Differences were found between resting and active motor map size, while map centrality and shape remained constant for both—supporting previous suggestions that motor map size scales linearly with muscle contraction level. More advanced map analysis methods may better characterize human motor maps for even greater utility in studies of motor system neurophysiology and plasticity.

## AUTHOR CONTRIBUTIONS

Cynthia K. Kahl: Conceptualization, methodology, validation, formal analysis, investigation, writing—original draft, writing—review & editing, visualization, project administration. Adrianna Giuffre: Conceptualization, methodology, validation, investigation, writing—review & editing, project administration. James G. Wrightson: Methodology, software, validation, formal analysis, writing—review & editing, visualization. Adam Kirton: Conceptualization, methodology, resources, writing—review & editing, supervision, funding acquisition, project administration. Elizabeth G. Condliffe: Conceptualization, methodology, resources, writing—review & editing, supervision.

Frank P. MacMaster: Conceptualization, resources, writing—review & editing, supervision, funding acquisition. Ephrem T. Zewdie: Conceptualization, methodology, software, validation, resources, writing—review & editing, supervision.

## CONFLICT OF INTEREST

The authors declare that they have no conflicts of interest or financial disclosures.

## Supporting information




Figure S1
