# Supplementary material for: Active versus resting neuro‐navigated robotic transcranial magnetic stimulation motor mapping
Source: Physiol Rep. 2022 Jun 24;10(12):e15346. doi: 10.14814/phy2.15346 (PMC9226845; doi:10.14814/phy2.15346)
Supplement: Supplementary file 1 — Figure S1 [file PHY2-10-e15346-s001.docx]

**Supplementary Material**

**Supplementary Figure 1. 2D resting and active motor maps**

| **A.**  **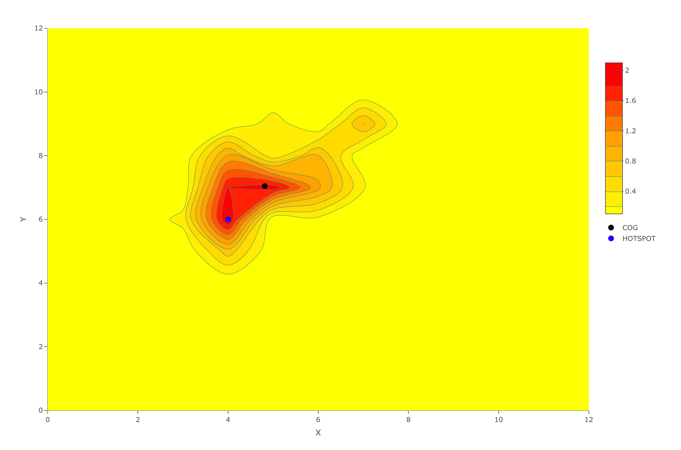** | **B.**  **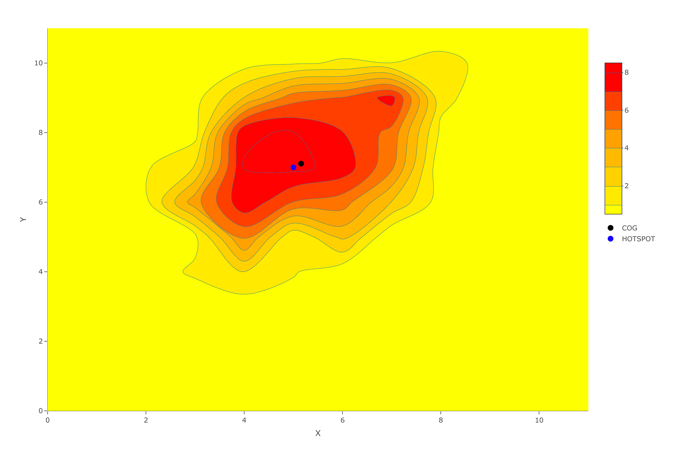** |
| --- | --- |

*Example 2D contour plots of* ***A.*** *resting and* ***B.*** *active motor maps.*
